# Systematic review and meta-analysis of randomised trials of perioperative outcomes comparing robot-assisted versus open radical cystectomy

**DOI:** 10.1186/s12894-016-0177-z

**Published:** 2016-09-23

**Authors:** Zhiyuan Shen, Zhongquan Sun

**Affiliations:** Department of Urology, Huadong Hospital, Fudan University, 221 West Yan’an Road, Shanghai, 200040 China

**Keywords:** Bladder cancer, Cystectomy, Meta, Robot

## Abstract

**Background:**

With the introduction of robotic surgery, whether the robot-assisted radical cystectomy (RARC) could reduce the perioperative morbidity compared with Open radical cystectomy (ORC) was unknown.

**Methods:**

Studies reported RARC were reviewed based on all randomized controlled trials (RCTs), which focused on the efficacy of RARC versus ORC.

**Results:**

Of the 201 studies from preliminary screening, four RCTs were included. By pooling these studies, there were significant differences in comparison of operative time (*p =* 0.007), estimated blood loss (EBL) (*p <* 0.001) and time to diet (*p <* 0.001) between the RARC group and ORC groups. There was no significant difference regarding perioperative complications (Clavien 2–5, Clavien 3–5), length of stay (LOS), positive surgical margins (PSM) and lymph node positive.

**Conclusion:**

This meta-analysis presented evidence for a benefit of EBL, time to diet, similar perioperative complications and oncological outcomes, but a longer operative time in RARC. It is noted that RARC was considered as a comparable surgical procedure to ORC.

## Background

In United States, approximately 74000 new cases of urinary bladder cancer with estimated 16000 deaths were expected in 2015 [1]. Open radical cystectomy (ORC) combined pelvic lymph node dissection (PLND) and urinary diversion (UD) is gold standard surgical intervention for high risk non-muscle invasive and muscle invasive bladder cancer, but accompanied with significant perioperative morbidity. In 2003, Menon reported the first case of robot-assisted radical cystectomy (RARC) [2]. With the introduction of robotic surgery, minimally invasive bladder cancer surgery set off a new climax with the promise of decreasing perioperative morbidity and mortality once again. Since then, a few prospective and retrospective studies had reported lower or comparable rates of complications, quicker recovery, and equivalent oncologic outcomes compared with ORC, however, which did not lead to a conclusive result [3–6]. Furthermore, these non-randomised researchs were accompanied with prominent selection bias. Although several meta-analyses regarding comparison of RARC with ORC had existed [7–11], these reviews incorporated a majority of non-randomized trials. Currently four randomized controlled trials had been publicated, therefore we conducted a systematical review of these literatures comparing surgical outcomes of RARC with those of ORC to provide powerful evidence.

## Methods

### Literature search

A systematic review of literatures was performed in Dec 2015. The electronic databases including PubMed, Embase, and the Cochrane Library were searched with restriction to English language. The following terms and their combinations were searched in [Title/Abstract]: cystectomy, cystectomies, cystoprostatectomy, bladder resection, robotic, robot, robot*, robot-assisted, and da Vinci. The related articles function was also used to broaden the search. Lists of references from the retrieved articles were manually searched to ensure as many studies as possible. When multiple reports describing the same population were published, the most recent or complete report was used.

### Inclusion criteria and exclusion criteria

All available randomized controlled trials comparing RRC and ORC were considered, in addition at least one outcome of interest was mentioned. Studies as follow were excluded: (i) prospective non-randomised trials or retrospective trials comparing RARC and ORC, editorials, letters to the editor, review articles, experimental animal studies, case reports, comments, and conference abstracts, (ii) no outcomes of interest were reported.

### Data extraction and outcomes of interest

Two reviewers independently selected studies for inclusion and extracted the following data: first author, year of publication, country, study interval, study design, indications for operation, number of patients who underwent RARC or ORC, rate of conversion from robot-assisted to open technique, matching criteria: age, gender, body mass index, American Society of Anesthesiologists (score), diversion type (conduit or neo-bladder), clinical stage, neoadjuvant chemotherapy, and outcomes of interest.

The primary outcomes were perioperative complication rates including intraoperative complications and postoperative complications classified according to the Clavien-Dindo grading system [12]. If sufficient data was available, perioperative complications were subdivided into 30d and 90d. The secondary outcome variables included operating time, estimated blood loss (EBL), number of patients receiving blood transfusion, length of stay (LOS), time to regular diet, positive margins, number of lymph nodes and pathologic stage. All disagreements were resolved by discussion until a consensus was reached. On condition that the data was incomplete, the corresponding authors were contacted.

### Methodological quality

An evaluation of the methodological quality of the eligible studies was performed according to the Cochrane handbook [13]. For risk of bias assessment, the selection bias, performance bias and detection bias, attribution bias, reporting bias and other potential sources of bias were assessed in each of the included studies. The intention-to-treat analyses were described in the majority of studies.

### Statistical analysis

The meta-analyses were conducted using Review Manager Version 5.3.

Dichotomous variables were presented as odds ratios (ORs) with 95 % confidence intervals (CIs), continuous variables as weighted mean differences (WMD) with 95 % CI. For studies that presented some continuous data as median and range values, the means and standard deviations were derived by statistical algorithms decribed by Wan et al. [14]. The *p*-Value was considered significant if <0.05. Statistical heterogeneity between studies was assessed using the chi-square test with *p <* 0.10 used for statistical significance. Statistical heterogeneity was also assessed using the I^2^ test: I^2^ values of 25 % (low), 50 % (medium), and 75 % (high). With I^2^ values of 50 % or less, heterogeneity was acceptable referring to Cochrane handbook and in case that high levels of heterogeneity with I^2^ values of 50 % or larger, we adopted a random-effects model.

## Results

### Characteristics of eligible studies

**Fig. 1:**
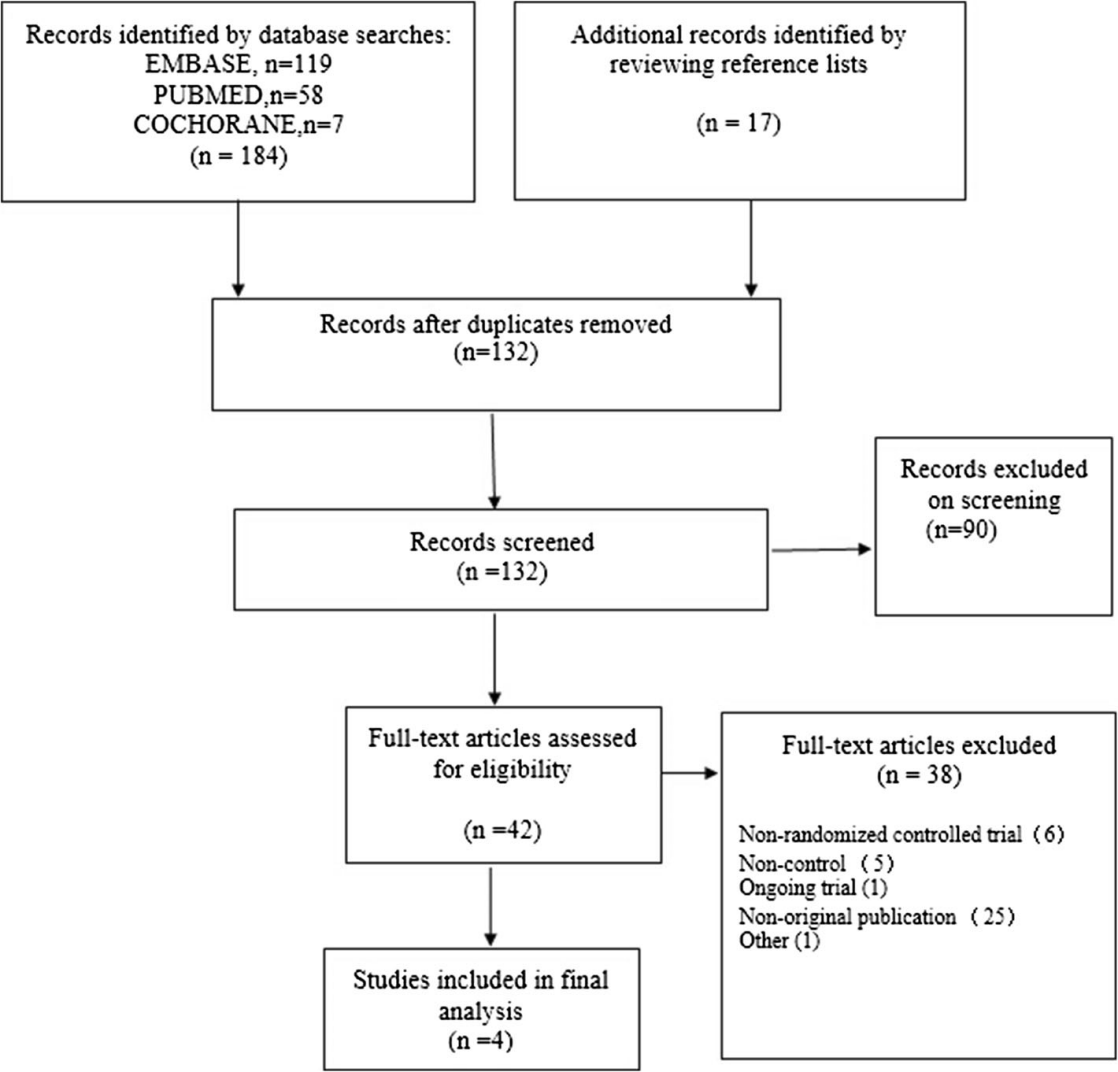
Flow diagram of studies identified, included, and excluded

A thorough review of the potentially relevant studies resulted in 201 articles of which 4 were selected in the final analysis including 239 cases (121 cases in RARC group and 118 controls in ORC group (Fig. 1). Participant characteristics were presented as follows (Table 1). All 4 RCTs scored level 2b. 3 RCTs reported extracorporeal urinary diversion method with the similar percentage of neobladder [15–17]. Only one patient converted to open surgery due to equipment failure [16]. Of these excluded studies, 5 lacked controls, 25 non-original publications, 6 non-randomized controlled trial, 1 ongoing trial, 1 shared overlapping populations with no outcomes of interest.

**Table 1:** Study characteristics

Publication	Country	Design	LOE	Case		Age (mean or median)		Conversion	Matching^a^	Neobladder		Urinary diversion method
				RARC	ORC	RARC	ORC			RARC	ORC	
Nix 2009	USA	RCT	2b	21	20	67.4	69.2	0	1,2,3,4,7,8	7	6	Extracorporeal
Parekh2012	USA	RCT	2b	20	20	69.5	64.5	0	1,2,3,4,6	NA	NA	NA
Bochner 2014	USA	RCT	2b	60	58	66	65	0	1,2,3,4,6,7,8	33	32	Extracorporeal
Khan 2015	UK	RCT	2b	20	20	68.6	66.6	1^b^	1,2,3,4,6,7,8	2	3	Extracorporeal

*RARC* robot-assisted radical cystectomy, *ORC* open radical cystectomy, *LOE* level of evidence, *RCT* randomized controlled trial, *NA* data not available

^a^Matching variables: 1 = age, 2 = gender, 3 = BMI, 4 = ASA, 5 = previous abdominal/pelvis surgery history, 6 = neoadjuvant chemotherapy, 7 = clinical stage, 8 = urinary diversion type

^b^due to equipment failure

**Fig. 2:**
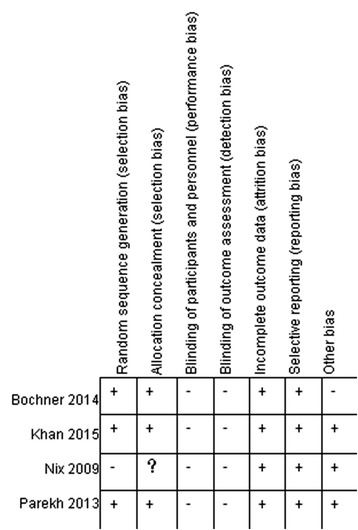
Risk of bias summary: review authors’ judgements about each risk of bias item for each included study

The risk of bias summary about each risk of bias item was available in Fig. 2. Nix et al. adopted inappropriate randomization, and allocation concealment could not be judged insufficient information. On account of different surgical approaches, blinding could not be achieved in four RCTs comparing RARC and ORC. Few data missing and ITT (intention-to-treat) analysis other than one RCT reduced attribution bias. Then all preestablished outcomes were entirely reported. Moreover, limited cases brought out relatively weaker Statistical power.

### Demographic and clinical characteristics

The mean or median age in the included studies acquired without sufficient standard variation for pooling data was quite close. Pooled data showed no difference in the male/female ratio or BMI. Clinical stage T4 was excluded, and no difference was found in distribution of T2-3 stage in three RCTs. There was also no difference in neochemotherapy applied in three studies. Likewise, the proportion of neobladder showed no difference in urinary conversion.

### Perioperative outcomes

**Fig. 3:**
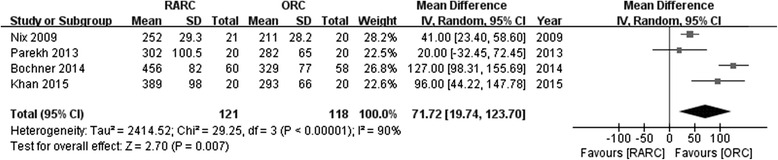
Forest plot and meta-analysis of operative time

**Fig. 4:**
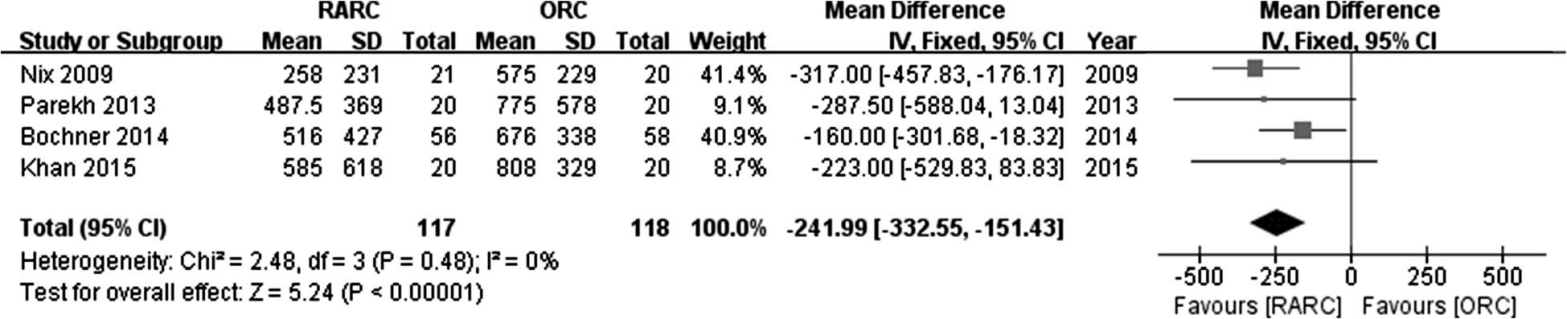
Forest plot and meta-analysis of estimated blood loss

Pooled data from 4 studies evaluated operative time and estimated blood loss showed significantly longer OP (WMD: 71.72; 95 % CI, 19.74 to 123.70; *p =* 0.007) (Fig. 3) and less EBL (WMD:−241.99; 95 % CI,−332.55 to−151.43; *p <* 0.00001) (Fig. 4) in the RARC than the ORC group.

**Fig. 5:**
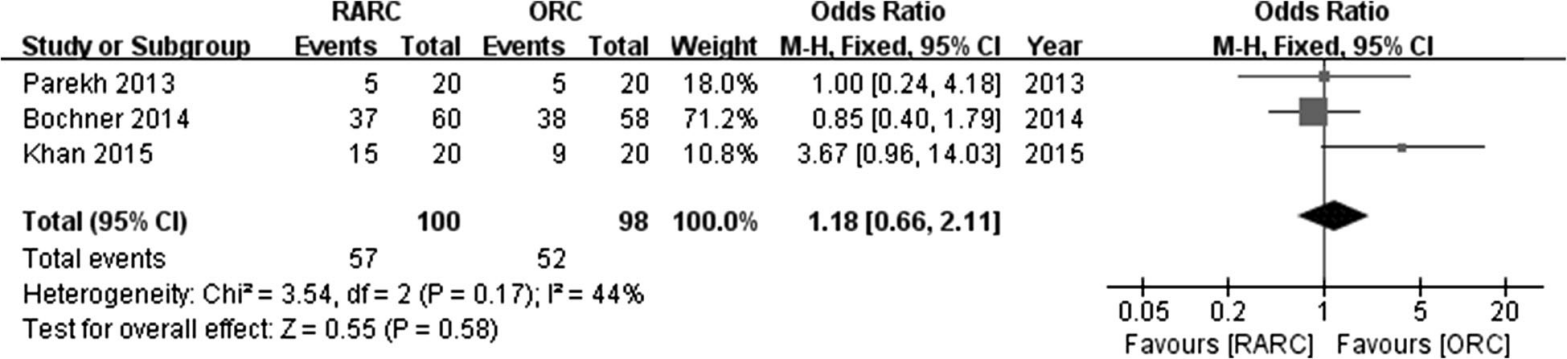
Forest plot and meta-analysis of perioperative complications (Clavien 2–5)

**Fig. 6:**
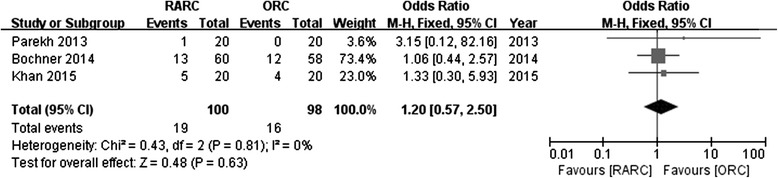
Forest plot and meta-analysis of perioperative complications (Clavien 3–5)

All RCTs compared complications using the Clavien system. Pooled data from 3 RCTs showed no significance in perioperative complications between RARC vs. ORC regarding to Clavien 2–5 (OR: 1.18; 95 % CI, 0.66 to 2.11; *p =* 0.58) (Fig. 5) or Clavien 3–5 (OR: 1.2; 95 % CI, 0.57 to 2.5; *p =* 0.63) (Fig. 6).

**Fig. 7:**
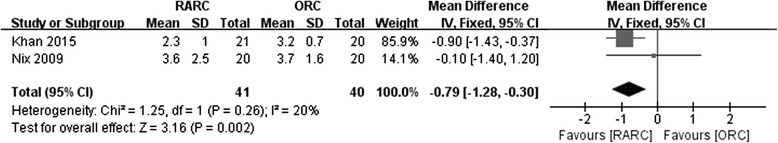
Forest plot and meta-analysis of time to flatus

**Fig. 8:**
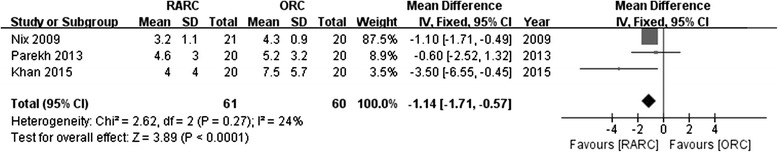
Forest plot and meta-analysis of time to regular diet

**Fig. 9:**
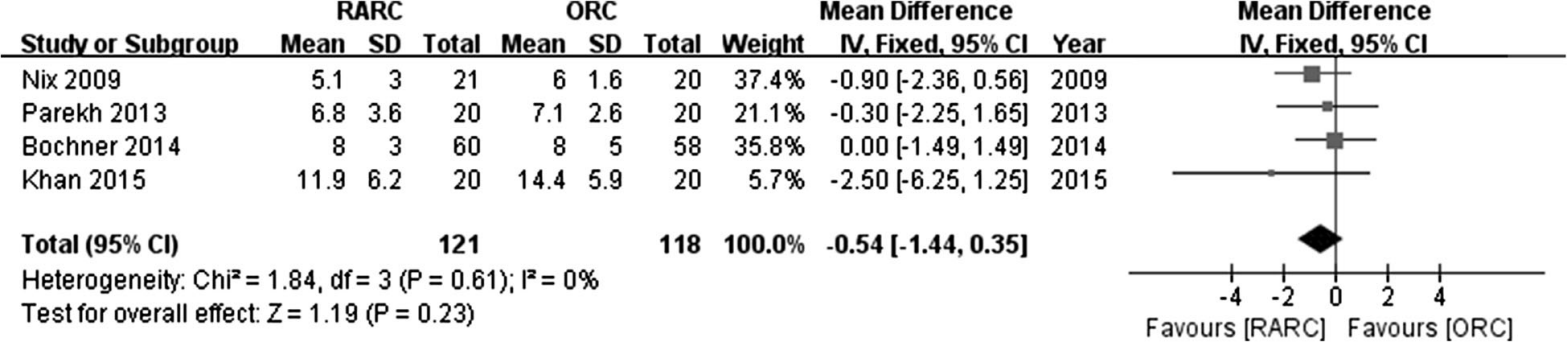
Forest plot and meta-analysis of length of stay

Data of time to flatus was extracted from 2 studies. Postoperative flatus was significantly shorter in RARC group (WMD:−0.79; 95 % CI,−1.28 to−0.30; *p =* 0.002) (Fig. 7). Likewise, time to regular diet from 3 studies was significantly shorter in RARC group (WMD:−1.14; 95 % CI,−1.71 to−0.75; *p <* 0.0001) (Fig. 8). There was no significant difference for length of stay between RARC and ORC from 4 RCTs (WMD:−0.54; 95 % CI,−1.44 to−0.35; *p =* 0.23) (Fig. 9).

### Pathologic outcomes

**Fig. 10:**
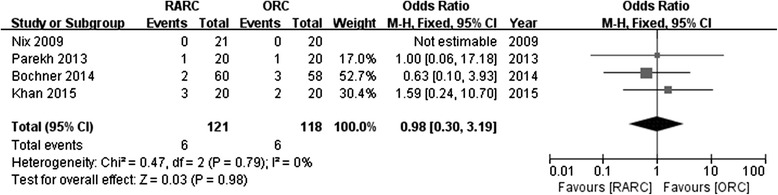
Forest plot and meta-analysis of positive surgical margin

**Fig. 11:**
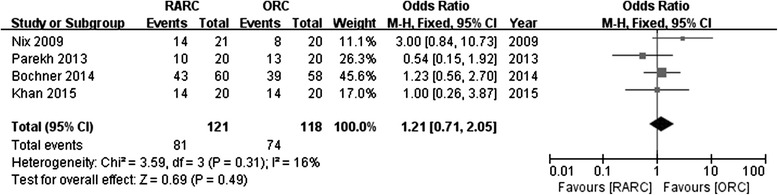
Forest plot and meta-analysis of pathological stage ≤ pT2

**Fig. 12:**
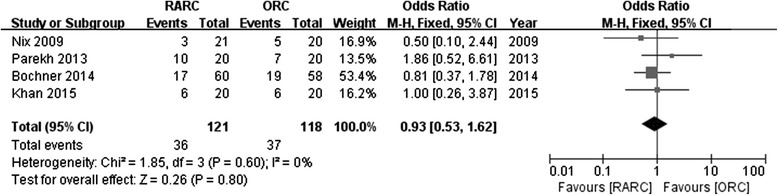
Forest plot and meta-analysis of pathological stage ≥ pT3

**Fig. 13:**
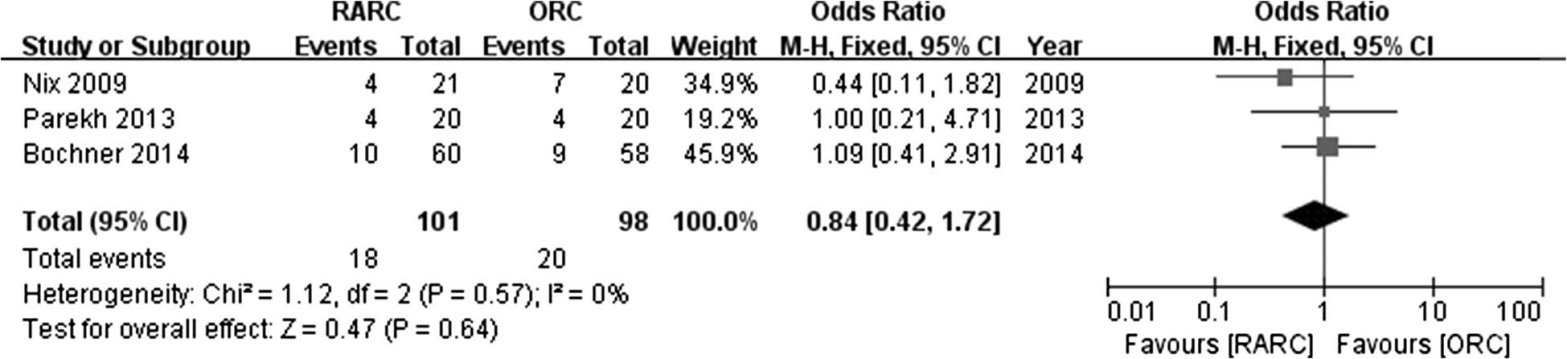
Forest plot and meta-analysis of lymph node positive

Four studies reported the rates of positive surgical margin. There was no significant statistical difference in PSM between RARC group and ORC group (OR: 0.98; 95 % CI, 0.30 to 3.19; *p =* 0.98) (Fig. 10). Pathological stage in detail was reported in 4 studies. No significance was found in part of ≤ pT2 (OR: 1.21; 95 % CI, 0.71 to 2.05; *p =* 0.49) (Fig. 11), or ≥ pT3 (OR: 0.93; 95 % CI, 0.53 to 1.62; *p =* 0.8) (Fig. 12). Data of lymph node positive was available in 3 studies. Similarly, there was no significant statistical difference between RARC group and ORC group (OR: 0.84; 95 % CI, 0.42 to 1.72; *p =* 0.64) (Fig. 13).

## Discussion

ORC with pelvic lymph node dissection still remains the gold standard approach for management of high grade non muscle-invasive and muscle-invasive bladder cancer. Notwithstanding, RARC became prevailing, especially in the U.S, which increased from 0.6 to 12.4 % of all RC cases from 2004 to 2010 [18]. Previous meta-analyses included both retrospective and prospective studies, which inevitably gave rise to selection bias. Fortunately, 4 RCTs comparing RARC and ORC could be achieved. For this reason, we conducted a meta-analysis with higher level of evidence. According to the analysis result, RARC had an advantage of less EBL and more rapid return to regular diet, but accompanied by longer operative time. Interestingly, this review showed similar rate of perioperative complications, which differed from what proposed by previous researchers. Besides, RARC was comparable with ORC in PSM, LOS.

On account of the 3 dimensional visual effect and elaborate operation, blood loss was much less in RARC group. Another important reason was the tamponade effect from the pneumoperitoneum used during RARC [19]. There is no doubt that it is the distinct advantage of RARC in terms of bleeding control, regardless of study types [5–8, 11, 15–17, 20]. Less EBL signified less blood transfusion. Unfortunately, only one RCT reported the rate of transfusion with no statistical difference [20], therefore pooling data could not be carried out.

Pooled data of operative time showed that RARC took longer time compared with ORC as with previous results [4, 21]. No matter docking Da Vinci system or conversion to open urinary diversion adopted was time-consuming. Parekh et al. reported the similar operative time for both surgical procedures, and uniquely depicted the definition of operative time (defined as incision to closure), however, the data of urinary diversion was not available [20]. In addition, a better comparison would be drawn in case that the time of radical cystectomy, PLND and UD could be set apart. Prior studies demonstrated that learning curve was significantly associated with shorter operative time [22, 23]. In this meta-analysis, 3 RCTs reported that surgeons performed approximately 50–110 RARCs to eliminate the impact of learning curve. However, Bochner et al. only gave a vague statement that surgeons were experienced in extensive robotic pelvic surgery experience.

Previous meta-analyses demonstrated a lower complications [7–11], however no significance was found in perioperative complications between RARC and ORC in this review. Less blood loss failed to bring about lower complications in RARC groups. Moreover, the study conducted by Bochner et al. terminated, because the primary objective that the rate of grade 2–5 complications would be 20 % lower for RARC compared with ORC was not reached. Hautmann et al. reported that the majority of complications after radical cystectomy were correlated with urinary diversion [24]. Recently, totally robotic-assisted radical cystectomy with intracorporeal diversion was increasingly adopted, which brought potential benefits to the patients. Ahmed et al. demonstrated a 32 % reduction in complications at 90d comparing open with robotic urinary diversion [25]. For overall complication rates, Koupparis et al. reported a trend to lower complication rate (31 % vs 48 %) in the RARC group vs ORC group. Introducing robotic-assisted radical cystectomy with intracorporeal diversion in the future RCTs might show the potential advantage for perioperative complications. Remarkably, extracorporeal urinary diversion was adopted in 3 RCTs, which might be the reason why lower perioperative complications could not be found in RARC group.

In overall surgical margin, we draw a conclusion of an equivalent in RARC group and ORC group. The total rate of PSM in RARC groups was 5 % (range, 0–15 %), which was similar to 6.8 % raised by the International Robotic Cystectomy Consortium [26]. Higher pathological T stage was significantly correlated with an increased likelihood of a positive margin [26]. In this review, pooled data of extravesical disease (≥ pT3) showed no significance difference. However, PSM could not be detailed according to stage (<pT2 vs > pT3) for insufficient data. Only Bochner et al. reported PSM in subgroup of patients ≥ pT3 (RARC versus ORC: 12 %, 16 %, *p =* 0.7) [15]. PLND served as an indicator of surgical quality of RC [27]. Parekh et al. reported an appearance of fewer median LNs in RARC with no significance. Nix et al. described a similar mean LNs between RARC and ORC. These data was insufficient for pooling data. Lymph node positive available in 3 studies was similar.

For long-term oncologic outcomes in RARC from International Robotic Cystectomy Consortium, after median follow-up of 67-months, 5 year recurrence-free survival, cancer-specific survival, and overall survival were 67 %, 75 %, and 50 %, respectively [28]. In this meta-analysis, only short term oncological outcomes were reported by Khan et al. that disease recurrence (11 %, 16 %) and disease-specific mortality (0 %, 5 %) was equivalent between RARC and ORC at 12 months.

With regard to LOS, although several retrospective studies and meta-analysis had demonstrated a significantly shorter LOS in RARC that may derive from the severe selection bias [4, 21, 29], but no significant difference was found in RARC group versus ORC group in our review, only modest trends of shorter LOS. The less bleeding and shorter time to regular diet had not brought about shorter LOS in our analysis. Above all, the similar complications might mainly contribute to longer hospital stay.

Outcomes of interest were mainly acquired, and incomplete data was depicted. The heterogeneity of variables was mostly low (I^2^ ≤ 50 %) except operative time (I^2^ = 90 %), for which random effects were taken.

Potential selection biases could be eliminated by the randomized trials, but surgeon’s experience might introduce an important confounder to results. Patients unable to bear the pneumoperitoneum and steep trendelenburg position were excluded in RARC, coupled with improper randomization method applied in Nix’s study. Hence, selection bias still existed.

## Conclusion

Four RCTs comparing RARC and ORC were included in this meta-analysis. Based upon analysis, a benefit of less EBL, shoter time to diet, similar perioperative complications and oncological outcomes, but a longer operative time could be seen in RARC group. We might draw a conclusion that RARC was a safe and effective surgical procedure noninferior to the open approach, meanwhile randomized controlled trial comparing RARC and ORC was feasible. Despite a rigorous methodological review, restrictions were exerted to draw a straightforward conclusion for limitations of the included studies and patients. Certainly, further prospective, multicentric and large sample randomized control trials should be undertaken to confirm our findings. It’s expected that Parekh et al. paved the way toward a phase III, multi-institutional, randomized trial that can offer more definitive answers.

## Abbreviations

Cis: Confidence intervals
EBL: Estimated blood loss
LOS: Length of stay
ORC: Open radical cystectomy
ORs: Odds ratios
PLND: Pelvic lymph node dissection
PSM: Positive surgical margins
RARC: Robot-assisted radical cystectomy
UD: Urinary diversion
WMD: Weighted mean differences
